# Land Cover Classification by Integrating NDVI Time Series and GIS Data to Evaluate Water Circulation in Aso Caldera, Japan

**DOI:** 10.3390/ijerph17186605

**Published:** 2020-09-10

**Authors:** Hiroki Amano, Yoichiro Iwasaki

**Affiliations:** 1 Liberal Arts Education Center, Kumamoto Campus, Tokai University, 9-1-1 Toroku, Higashi-ku, Kumamoto 862-8652, Japan; ah069881@tsc.u-tokai.ac.jp; 2 Department of Electrical Engineering and Computer Science, Faculty of Industrial and Welfare Engineering, Tokai University, 9-1-1 Toroku, Higashi-ku, Kumamoto 862-8652, Japan

**Keywords:** NDVI, satellite image, Landsat, GIS, groundwater recharge, water circulation

## Abstract

Grasslands in Aso caldera, Japan, are a type of land cover that is integral for biodiversity, tourist attractions, agriculture, and groundwater recharge. However, the area of grasslands has been decreasing in recent years as a result of natural disasters and changes in social conditions surrounding agriculture. The question of whether the decrease in spring water discharge in Aso caldera is related to the decrease in grasslands remains unanswered. To clarify this relationship, a water circulation model that considers land covers with different hydrological features is needed. In this study, by integrating Normalized Difference Vegetation Index (NDVI) time series and Geographic Information System (GIS) data, we generated land cover maps from the past (in 1981 and 1991) to the present (in 2015 and 2016), before and after the 2016 Kumamoto earthquake, and then for the future (in the 2040s); these maps formed the dataset for building a water circulation model. The results show that the area of grasslands, which are reported to have a higher groundwater recharge rate than that of forests, in 2016 had decreased to 68% of the area in 1981 as a result of afforestation and transformation into forests, as well as landslides induced by the earthquake. The area of grasslands is predicted to further drop to 60% by the 2040s. On the other hand, the area of forests (conifers and hardwoods) in 2016 had increased by 119% relative to that in 1981 because of the transformation of grasslands into forests, although these areas decreased as a result of landslides due to the 2016 Kumamoto earthquake. Quantification of groundwater recharge from grasslands and forests using the land cover maps generated for 1981, 1996, 2015, and 2016 shows that the annual increase in precipitation in these years significantly affected groundwater recharge; these effects were greater than those associated with the type of land cover. Thus, the groundwater recharge increased, despite the decrease in grasslands. However, when constant precipitation was assumed, the groundwater recharge presented a decreasing trend, indicating the importance of maintaining and conserving grasslands from the viewpoint of groundwater conservation.

## 1. Introduction

Aso caldera, located in the northeastern part of Kumamoto Prefecture, Japan, is one of the largest in the world. It was formed incrementally over a period of 300 kyr by the eruption of four pyroclastic flow and fall deposits (Aso 1–4) [1]. Aso caldera and its surrounding area are covered by the largest scale grasslands in Japan, whose area reaches approximately 220 km^2^ [2]. Grasslands are an important environment for various living things including rare species registered as endangered species [3]. Because of its majestic landscape of volcanoes and grasslands, Aso region was designated as a national park in 1934. These landscapes act as tourism resources and attracted over 18 million tourists in 2004 [3]. Precipitation in this vast land, including the outer rim, is about twice the mean value for Japan. This precipitation is recharged into the ground and gushes out from over 1500 springs, which form the source of the six largest rivers in Kyushu Island [3]. The basin of the six rivers has a vast area of 8724 km^2^, in which 2.3 million people live [3]. The rivers are important water resources for uses such as irrigation and domestic water. For example, the Shirakawa River, which flows from within Aso caldera to Kumamoto City, which is the capital city of Kumamoto Prefecture, has been used as irrigation water for paddy fields in the mid-stream area. Water that infiltrates paddy fields becomes an essential part of groundwater that covers almost 100% of domestic water use in the Kumamoto City whose population is about 740 thousand. Kumamoto city, where a large number of populations relies on groundwater, is the only city in Japan and rare in the world [4]. Thus, the Aso region is important for water resources and circulation in northern and central Kyushu Island. However, this region has been stressed by changes in the agriculture system, the 2012 northern Kyushu heavy rain, and the 2016 Kumamoto earthquake. In particular, the grasslands, which are important for biodiversity, tourist attractions, and groundwater recharge and have long been maintained by human activities, have been rapidly disappearing as a result of transformations of grasslands into forests. In recent years, there have been concerns that the discharge of spring water may be decreasing as a result of the decrease in grasslands. This is because the annual groundwater recharge rate (mm/year) of grasslands is higher than that of forests [5]. Actually, the spring discharge in the Shirakawa spring source, which was reported to be 60 m^3^/min before 2000 [6], decreased to about two-thirds of that value in 2019 (The Asahi Shimbun, 13 May 2020; morning newspaper). This is a significant problem in Aso caldera, where spring water has been used as a resource for agricultural and domestic water use. However, the cause–effect relationship between the decreases in spring discharge and that in grasslands is uncertain. Similar to Ezu Lake in the Kumamoto Plain, where the discharge of spring water has decreased because of the decrease in paddy fields, it is possible that the decline in discharge is related to the area change in paddy fields. In order to maintain and restore the discharge of spring water, that is, to preserve groundwater, it is crucial to investigate the relationship between land cover changes and spring discharge.

To clarify the impact of land cover changes due to social conditions and natural disasters on the water circulation, it is necessary to build a model that effectively represents the features of the study area. The key factors here are various hydrogeological parameters, such as precipitation, evapotranspiration, hydraulic conductivity of soil (ground), the infiltration rate of the soil surface, and water requirement rates of paddy fields. Of these parameters, the evapotranspiration and infiltration rates are significantly different depending on the land cover. When annual precipitation is 3000 mm, annual evapotranspiration in grasslands and forests is about 800 mm and 1400 mm, respectively [7]. The infiltration rate was shown to be higher in forests than in grasslands (artificial conifers: 260.2 mm/h; natural hardwoods: 271.6 mm/h; natural grasslands: 143.0 mm/h; artificial grasslands: 107.3 mm/h) [8]. Furthermore, a characteristic of water in paddy fields is that it continuously infiltrates into the ground during periods in which the fields are flooded. Naturally, even for the same land cover, the values of hydrological parameters vary from place to place. Therefore, the location and area of each land cover type with different hydrological features are also important data for constructing a model.

Geographic Information System (GIS) data related to land cover are available through various organizations in Japan. Land cover subdivision mesh data [9], actual vegetation maps [10], and high-resolution land use and land cover maps [11] can be obtained from the corresponding websites for free. The land cover subdivision mesh is the only data that can be used to track land cover changes in detail because eight datasets of this information have been released from 1976 to 2016. However, the data do not contain classifications of grasslands. Although actual vegetation maps classify vegetation in detail, there are only two data sources: a 1/50,000 scale map from about 40 years ago and a more recent 1/25,000 scale map. These high-resolution land use and land cover maps have resolutions of 10 m and 30 m, but there are only two that represent the average land cover in recent years (2006–2011 and 2014–2016). A comparison of these maps with satellite images reveals that they do not necessarily represent the actual land cover. Naturally, such big data are not updated every year, so land cover changes on short time scales, such as before and after an earthquake, cannot be captured. Therefore, the main objective of this study is to generate land cover maps from the past to recent years, before and after the 2016 Kumamoto earthquake, in order to construct a water circulation model that is suitable for Aso caldera and to then quantify the groundwater recharge volume from forests, grasslands, and paddy fields, which form the main groundwater recharge area. In addition, one of our goals is to predict the transition of grasslands to forests in the future. The Normalized Difference Vegetation Index (NDVI), which is the index representing the activity of vegetation, is effective for the creation of land cover maps and the assessment of land cover changes [12,13,14,15,16]. The NDVI is also effective for extracting the locations of landslides induced by earthquakes [17,18]. Therefore, we attempted to generate land cover maps by integrating a characteristic NDVI time series data corresponding to land cover in Aso [19] and the GIS data mentioned above. Then, in order to understand the importance of grasslands for groundwater recharge, we quantified its changes due to the transformation of grasslands into forests by using the generated land cover maps and the constructed water balance model.

## 2. Materials and Methods

### 2.1. Study Area

**Figure 1:**
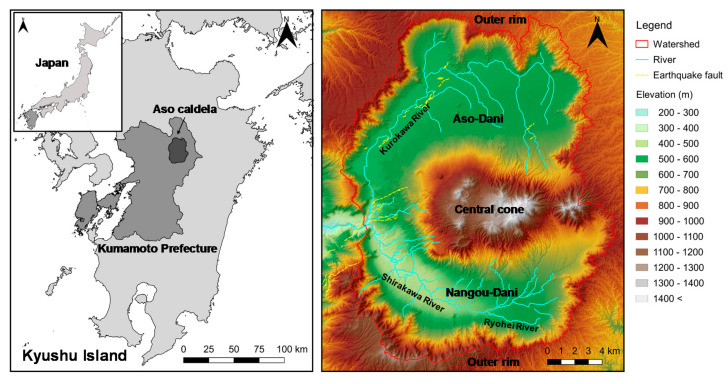
Location map of the study area, including Shirakawa and Kurokawa River watersheds.

Aso caldera, located in the center of Kyushu Island, is the second-largest caldera in Japan (Figure 1). The area reaches 25 km north–south and 18 km east–west. Surrounded by the steep cliffs of the outer rim, the central cones, with altitudes of 1000–1600 m, divide the Aso caldera into north and south. The northern part is called Aso-Dani, and the southern part is called Nangou-Dani. The annual average precipitation and temperature from 1950 to 2014 in the central cones (Mt. Aso observatory) were 3038 mm and 9.7 °C, respectively [20]. Most of the hillside rivers are dried up, and direct runoff occurs when continuous precipitation is above 100–200 mm [21]. Such precipitation is especially likely to occur during the rainy season (June and July), but it typically occurs only a few times a year. Thus, most of the precipitation infiltrates into the ground. 

### 2.2. Satellite Images

Satellite images from Landsat-1 (launched in 1972) to Landsat-8 (which is currently in operation) are effective data for tracking temporal changes in land covers, although they have different resolutions. Therefore, in order to classify land covers, 12 satellite images from 1981 to 2017 were selected and downloaded from the LandsatLookViewer of United States Geological Survey (USGS) [22]. These data were projected to zone 52N in the Universal Transverse Mercator, which is often applied in Japan. Table 1 shows the profiles of the 12 satellite images. Landsat-8 Operational Land Imager (OLI) multispectral bands 1–7 and 9 have a resolution of 30 m. The resolutions of Landsat-5 Thematic Mapper (TM) multispectral bands 1–5 and 7 are 30 m as well. However, Landsat-2, 3 Multispectral Scanner (MSS) spectral bands 4–7 have a larger resolution of 80 m. In this study, we generated land cover maps for 1981, 1996, 2015 (before the earthquake), 2016 (after the earthquake), and the 2040s. The satellite images from Season II to Season IV were mainly applied to generate land cover maps up to 2016. This is because the NDVI of each land cover undergoes characteristic changes in these seasons [19]. In the case of paddy fields, the NDVI decreases significantly in Season III when planting begins. Aso caldera is often covered by clouds due to its topographic features. Although the analysis only requires satellite images from three time points, it was difficult to obtain them throughout the year. Thus, the satellite images of the missing season were complemented by those of the other years. The satellite images in Seasons I and V were used to predict the forestation of grasslands in the 2040s, as mentioned below. In the Aso region, according to Yamamoto et al. [23], woody species have increased and now occupy half of the ground surface in grasslands where open burning has been not performed for the past 30 years. Therefore, we set the 2040s, which is about 30 years after the 2016 Kumamoto earthquake, as the target year for the prediction.

The NDVI was calculated using the reflectance in the red band and near-infrared band:

(1) NDVI=(NIR−R)/(NIR+R)

where *R* is the reflectance of the red band, and *NIR* is the reflectance of the near-infrared band. As summarized in Table 2, in Landsat-8 OLI, band-4 corresponds to *R*, and band-5 corresponds to *NIR*. In Landsat-5 TM, band-3 corresponds to *R*, and band-4 corresponds to *NIR*. Landsat-2, 3 MSS has one red band (band-5) and two kinds of near-infrared bands with different wavelengths (band-6: 700–800 nm; band-7: 800–1100 nm). Thus, the NDVIs that were calculated by using these bands were adjusted by applying the following equation [24]:

(2) TM43=−0.0064+0.7097MSS65+0.3564MSS75

where TM43 is the adjusted NDVI, MSS65 is the NDVI calculated by using band-6 and -5 of Landsat-2, 3, and MSS75 is the NDVI calculated by using band-7 and -5 of Landsat-2, 3.

### 2.3. Land Cover Classification

**Figure 2:**
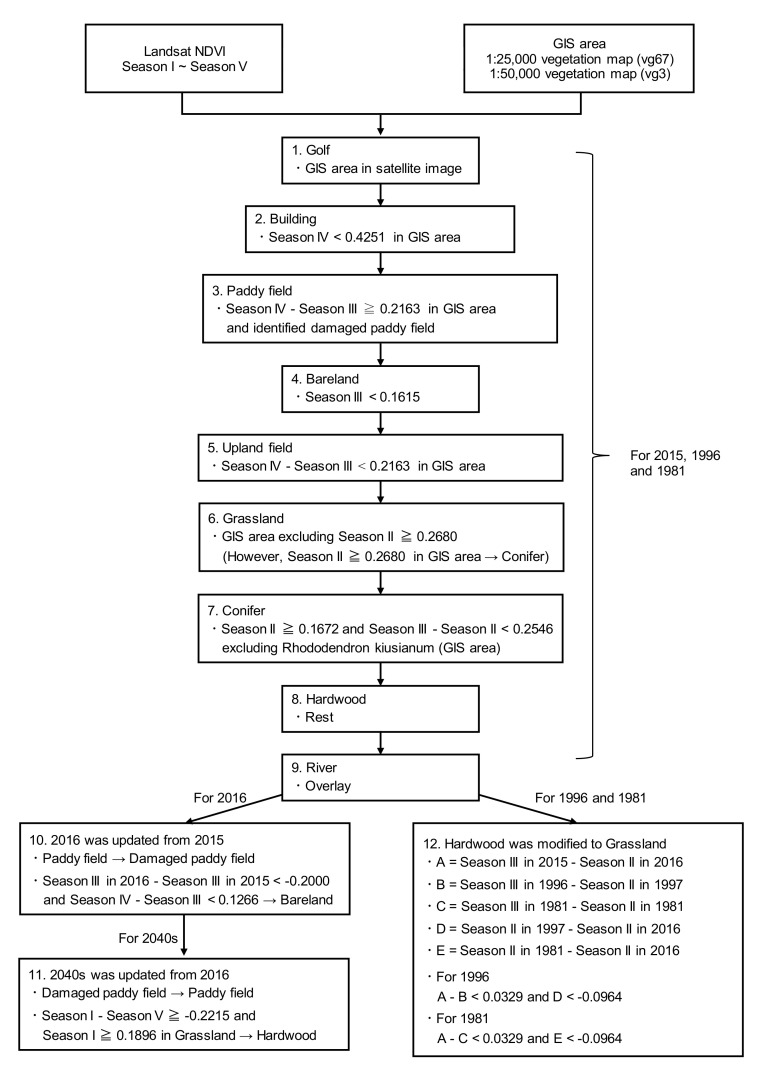
Flowchart of land cover classification.

In this study, the Shirakawa and Kurokawa River watersheds in Aso caldera (Figure 1) were classified into 10 categories: Grassland, Paddy field, Conifer, Hardwood, Upland field, Bare land, Damaged paddy field, Building (including plastic greenhouse), Golf, and River (water area). Among these, plastic greenhouses were identified by reading a SPOT-6 satellite image taken on 3 November 2016. The locations of damaged paddy fields were identified by using an account book of paddy fields and the Agriculture Land Information System [25]. The account book of paddy fields is a register that reports the address, area, and cultivated crops (e.g., rice for food, whole crop silage, soybean, tomato, buckwheat) for each field. The Agriculture Land Information System identifies the location of each field by its address. The river data were created by modifying the data of the 1/25,000 vegetation map [10] by using digital national land information [9], Google Maps, and Landsat satellite image. The other land covers were extracted in the order shown in the flowchart in Figure 2. The GIS area in Figure 2, which was used as a mask, basically indicates the limits in the 1/25,000 vegetation map (hereafter vg67) or 1/50,000 vegetation map (hereafter vg3) of the category to be extracted. The land cover maps of vg67 and vg3 were constructed through field surveys and interpretation of satellite images [10]. The surveys for vg67 started in 1999 and are still ongoing. The surveys for vg3 were performed from 1978 to 1979 and 1983 to 1987. Steps 1–8 are common to 1981, 1996, and 2015. The map for 2016 proceeds from step 9 to 10 and further to step 11 for the 2040s. On the other hand, the maps of 1981 and 1996 proceed from step 9 to 12. The flowchart in Figure 2 supplements the explanation that follows.

(1). The Golf area was modified by comparing vg67 and the satellite image of the target year.
(2). The GIS area that was used to extract the Building area of 2015 was from vg67. The Building classification result for 2015 was used as the GIS area for 1996. Further, the Building classification result for 1996 was used as the GIS area for 1981.
(3). The GIS area that was used to mask the Paddy field area was the paddy and upland areas of vg67 for 2015 and 1991 and vg3 for 1981. This is because the vegetation map may actually indicate paddy fields, even if they are upland fields. In addition, the Damaged paddy field area was added as Paddy field area for 2015 to ensure consistency with the Damaged paddy field area for 2016.
(4). Bare land was extracted only by NDVI without the GIS area.
(5). The GIS area that was used to mask Upland field was the paddy and upland areas of vg67 for 2015 and 1991 and vg3 for 1981. The reason for using the GIS area of paddy and upland areas as the mask is to maintain consistency with the Paddy field extracted in step 3.
(6). The NDVI of burned grasslands in Season II has a lower value than that of other land covers [26]. However, the NDVI of unburned grasslands is similar to that of hardwoods, so they are indistinguishable. Therefore, the grasslands of vg67 were applied to Grassland for 2015. Since some parts of grasslands in vg67 were confirmed to have changed to conifers through plantation, these areas were adjusted to Conifer by the NDVI threshold value. Similar to the step for Building (step 2), the classification result for Grassland for 2015 was used as the GIS area for 1996. Furthermore, the classification result for Grassland for 1996 was used as the GIS area for 1981.
(7). Conifer was extracted using two threshold values. However, in order to modify the results around the central cone, areas of *Rhododendron kiusianum* were excluded. For 2015 and 1991, vg67 was used for *Rhododendron kiusianum* areas, and vg3 was applied for 1981. The threshold value of 0.2546 was modified to 0.3265 for 1981 in order to improve the validity of the classification result, as mentioned later.
(8). The remaining areas that were not classified into any land covers in steps 1–8 were Hardwood.
(9). The River area created by the above method was overlaid.
(10). The 2016 land cover map was generated by updating the 2015 land cover map with the Damaged paddy field and Bare land resulting from the landslide of the 2016 Kumamoto earthquake. For Bare land induced by landslides, the regions in which the difference between the NDVI of Season III in 2016 and that of Season III in 2015 was less than −0.2 were first extracted [17,18]. If the extracted area is actually bare land, then the vegetation cannot be recovered easily. Therefore, with the above method, the extracted area that represents the NDVI change shown in Figure 2 was identified as Bare land due to landslides.
(11). The future prediction assumed that the damaged agriculture had recovered, and Damaged paddy field for 2016 was updated to Paddy field. Then, it was assumed that the unburned grasslands had changed to hardwoods. The unburned grasslands were updated to hardwoods by using the characteristic NDVI of unburned grasslands, as indicated by Yasunaka et al. [26].
(12). The future Grassland area was determined as described in step 6. Thus, considering the transition from grasslands to hardwoods, the Grassland in the past should be naturally larger than that in the future. Therefore, we added a procedure to modify Hardwood to Grassland according to the 1996 and 1981 land cover maps. The relationship between NDVI changes from Season II to Season III for burned grasslands and hardwoods is ‘burned grasslands > hardwoods’. On the other hand, the relationship between NDVIs for each land cover in Season II, just after open burning, is revealed to be ‘burned grasslands < hardwoods’. Step 12 applied these relationships.

### 2.4. Validity Assessment of Land Cover Map

We assessed the validity of the classification results by comparing the area of each category between the 2015 land cover map and vg67 and between the 1981 land cover map and vg3. The coefficient of determination *R*^2^ was chosen for verification. The Geoprocessing tool of ArcGIS Pro2.4 (ESRI) was used to calculate the area of each category. The coefficient of determination *R*^2^ is defined as below:

(3) $$R^{2}=1-\frac{\sum_{i=1}^{n}{\left(y_{i}-f_{i}\right)}^{2}}{\sum_{i=1}^{n}{\left(y_{i}-\bar{y}\right)}^{2}}$$

where *n* is the number of land cover categories, *y_i_* is the area of each land cover, *ȳ* is the averaged area, and *f_i_* is the estimated area computed by using the regression formula.

### 2.5. Estimation of Potential Groundwater Recharge

In this study, we quantified the impact of land cover changes from grasslands, the preservation of which is desirable, to forests (conifers and hardwoods) on groundwater recharge. According to Shimotsu [21], the water balance near the soil surface of the recharge area can be expressed as follows:

(4) P=Q+E+D

where *P* is precipitation, *Q* is groundwater recharge, *E* is evapotranspiration, and *D* is direct runoff. According to Zhang [7], evapotranspiration in forests and grasslands can be expressed as follows:

(5) $$ET_{F}=\left(\frac{1+2\frac{1410}{P_{a}}}{1+2\frac{1410}{P_{a}}+\frac{P_{a}}{1410}}\right)P_{a}$$

(6) $$ET_{G}=\left(\frac{1+0.5\frac{1100}{P_{a}}}{1+0.5\frac{1100}{P_{a}}+\frac{P_{a}}{1100}}\right)P_{a}$$

where *ET_F_* is the annual evapotranspiration in forests, *ET_G_* is the annual evapotranspiration in grasslands (mm), and *P_a_* is the annual precipitation (mm). In the watershed of the study area, Shimotsu [21] reported that direct runoff occurs when continuous precipitation is above 100–200 mm. If continuous precipitation is defined as precipitation of 0 mm that continues for 3 h, it is extremely rare that it exceeds 100–200 mm. This could occur a few times a year. Therefore, we ignored the direct runoff in this study and treated groundwater recharge as potential groundwater recharge.

Then, we evaluated potential groundwater recharge from paddy fields by using the following equation [27]:

(7) $$Q_{p}=I_{b}n_{b}+I_{b}\frac{n_{m}}{2}+I_{a}n_{a}+I_{a}\frac{n_{i}}{2}$$

where *Q_p_* is potential groundwater recharge from paddy fields (mm), *I_b_* and *I_a_* are infiltration rates before and after mid-summer drainage (mm/day), *n_b_* and *n_a_* are flooding days before and after mid-summer drainage, *n_m_* is mid-summer drainage days, and *n_i_* is intermittent irrigation days. In Aso caldera, rice for food and Whole Crop Silage (WCS) is cultivated in paddy fields. Since WCS is harvested when it is still green, the flooded days for WCS are shorter than that for rice grown for food. However, WCS could not be distinguished by the obtained satellite images. In addition, the area of WCS was smaller than that of rice grown for food according to the 2016 account book of paddy fields. Thus, referring to the irrigation days for rice grown for food, we set *n_b_* to 42, *n_m_* to 10, *n_a_* to 31, and *n_i_* to 36 [27]. The infiltration rate can be expressed as the difference between the water requirement rate and evapotranspiration. Water requirement rates before mid-summer drainage are 4.4–102.0 and 12.0–127.8 mm/day in Aso-Dani and Nangou-Dani, respectively [27,28]. After mid-summer drainage, water requirement rates are 13.3–235.2 and 37.1–88.6 mm/day in Aso-Dani and Nangou-Dani, respectively [27,28]. Thus, the evapotranspiration on the dates for which water requirement rates were investigated was subtracted from these water requirement rates. The evapotranspiration on each date was calculated by using the Hamon method [29]:

(8) $$ET_{d}=0.1651\times Ld\times RHOSAT\times KPEC(ET_{d}=0\mathrm{when}T<0)$$

where *ET_d_* is daily evapotranspiration (mm/day), and *Ld* is daytime length, which is the time from sunrise to sunset in multiples of 12 h. *RHOSAT* is saturated vapor density (g/m^3^) at the daily mean air temperature (*T*), where

(9) RHOSAT=216.7×ESAT/(T+273.3)

(10) ESAT=6.108×EXP(17.26939×T/(T+237.3))

In the above equations, *T* is the daily mean air temperature (°C), *ESAT* is saturated vapor pressure (mb) at the given *T*, and *KPEC* is the calibration coefficient, which was set to 1.2 in this study. Then, averaged infiltration rates of 35.5 and 64.6 were assigned to *I_b_* and *I_a_*, respectively. Simply put, to evaluate groundwater recharge by practicing paddy field agriculture, the analysis in this study did not consider recharge from paddy fields due to precipitation that occurs when rice for food or WCS is not being cultivated. 

Finally, groundwater recharge from the building area, which is an important context as a non-infiltration area, was evaluated by the following equation [30]:

(11) $$Q_{b}=P_{d}-S_{d}-ET_{d}\;(Q_{b}=0\mathrm{when}P_{d}<S_{d}+ET_{d})$$

where *Q_b_* is potential groundwater recharge from the building area (mm/day), *P_d_* is daily precipitation (mm/day), and *S_d_* is daily surface runoff (mm/day). The daily surface runoff can be expressed as below:

(12) $$S_{d}=CP_{d}$$

where *C* is the runoff coefficient, which was set to 0.95 in this study [30]. For precipitation, daytime length, and temperature, the averaged values of Aso Otohime and Takamori observatories [20], from which all data for the watershed can be obtained for 1981, 1996, 2015, and 2016, were used.

## 3. Results and Discussions

### 3.1. Validity of Land Cover Map

**Figure 3:**
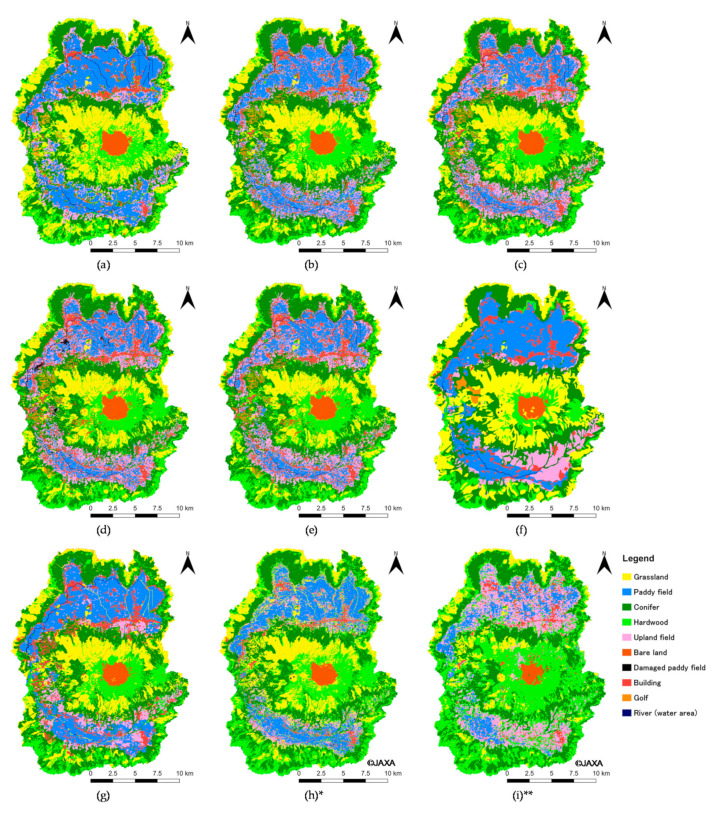
Land cover classification results and vegetation maps: (**a**) 1981, (**b**) 1996, (**c**) 2015, (**d**) 2016, (**e**) 2040s, (**f**) vg3, (**g**) vg67, (**h**) JAXA2006–2011 *, (**i**) JAXA2014–2016 **. * Provided by high-resolution land use and land cover 10 m resolution map of Japan [2006–2011] (ver.16.09: 10 categories) (Japan Aerospace Exploration Agency); ** Provided by high-resolution land use and land cover 30 m resolution map of Japan [2014–2016] (ver.18.03: 10 categories) (Japan Aerospace Exploration Agency).

**Figure 4:**
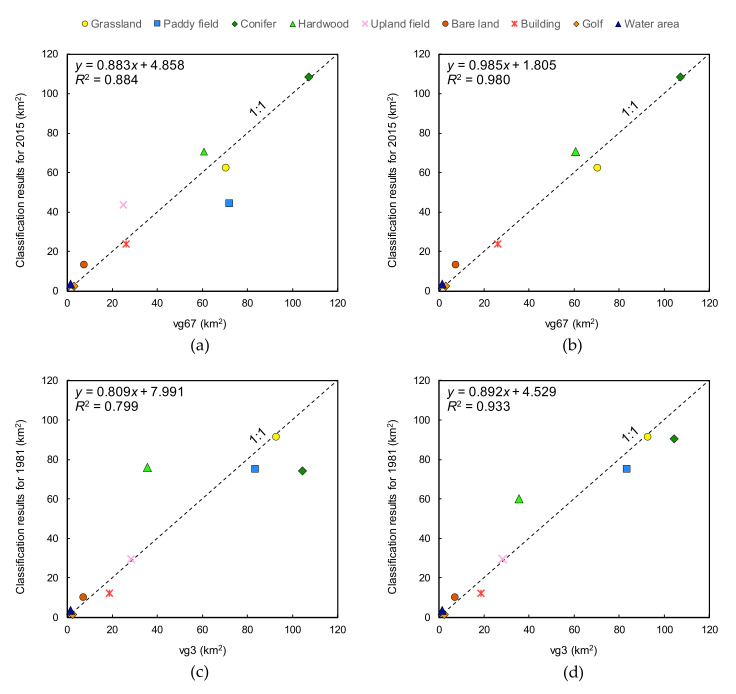
Relationships between vegetation maps and classification analysis results: (**a**) 2015 against vegetation map; (**b**) 2015 against vegetation map, except for paddy and upland fields; (**c**) 1981 against vegetation map; (**d**) modified 1981 against vegetation map.

Figure 3 shows the results of land cover classifications, vg3, vg67, and two Japan Aerospace Exploration Agency (JAXA) land cover maps. Next, Figure 4 represents the relationships of the area for each category between vg3, vg67, and the classification results. The distributions for each category of the 2015 classification result (Figure 3c) and vg67 (Figure 3g) are almost the same. However, there is a significant difference in Paddy and Upland fields (Figure 4a), and the *R*^2^ is 0.884. For the Paddy field, the area in the classification result is about 25 km^2^ smaller than the area in vg67. The area of the Paddy field calculated from the account book of paddy fields is 43.17 km^2^, so the classification result of 44.66 km^2^ is considered to be appropriate. For the Upland field, the area in the classification result is about 20 km^2^ larger than the area in vg67. The area of Upland fields calculated from the account book of paddy fields is 16.58 km^2^, whereas the classification result (43.64 km^2^) is much larger. This is mainly because the area of Paddy field in vg67 was classified as Upland field in the analysis; additionally, the abandoned fields were classified as Upland fields, although this category was not listed in the account book of paddy fields. Given the above, the classification results of Paddy and Upland field areas were considered to be valid. After excluding these categories, *R*^2^ increased to 0.980 (Figure 4b). 

For 1981, with the use of a threshold value of 0.2546, as shown in Figure 2, the differences in Conifer and Hardwood are significantly large, and *R*^2^ is 0.799 (Figure 4c). Therefore, in order to improve this result, the threshold value of 0.2546 was changed to 0.3265. The value of 0.2546 is the average of the NDVI changes from Season II to Season III for Conifer and Hardwood. The value of 0.3265 is the NDVI change from Season II to Season III for Hardwood. Consequently, *R*^2^ is improved to 0.933 (Figure 4d), and the distributions are almost the same (Figure 3a,f). However, there are differences for Paddy and Upland fields in the eastern parts of Nangou-Dani. Irrigation water is mainly supplied to the paddy fields from the springs on the right bank of the Shirakawa River and from five irrigation canals that receive water from Shirakawa and Ryohei Rivers on the left bank. The shortage of irrigation water is covered by pumping groundwater, and the number of pumping wells in Nangou-Dani reached about 600 in 1984 [31], although this is slightly after 1981. Although there are springs and irrigation canals, it is difficult to imagine that 600 wells had been installed for the paddy fields shown in vg3 (Figure 3f). In addition, we actually confirmed a number of wells in the upland fields of vg3 during the field survey. Thus, it is reasonable to consider the paddy fields to be distributed as shown in the analysis result (Figure 3a).

**Figure 5:**
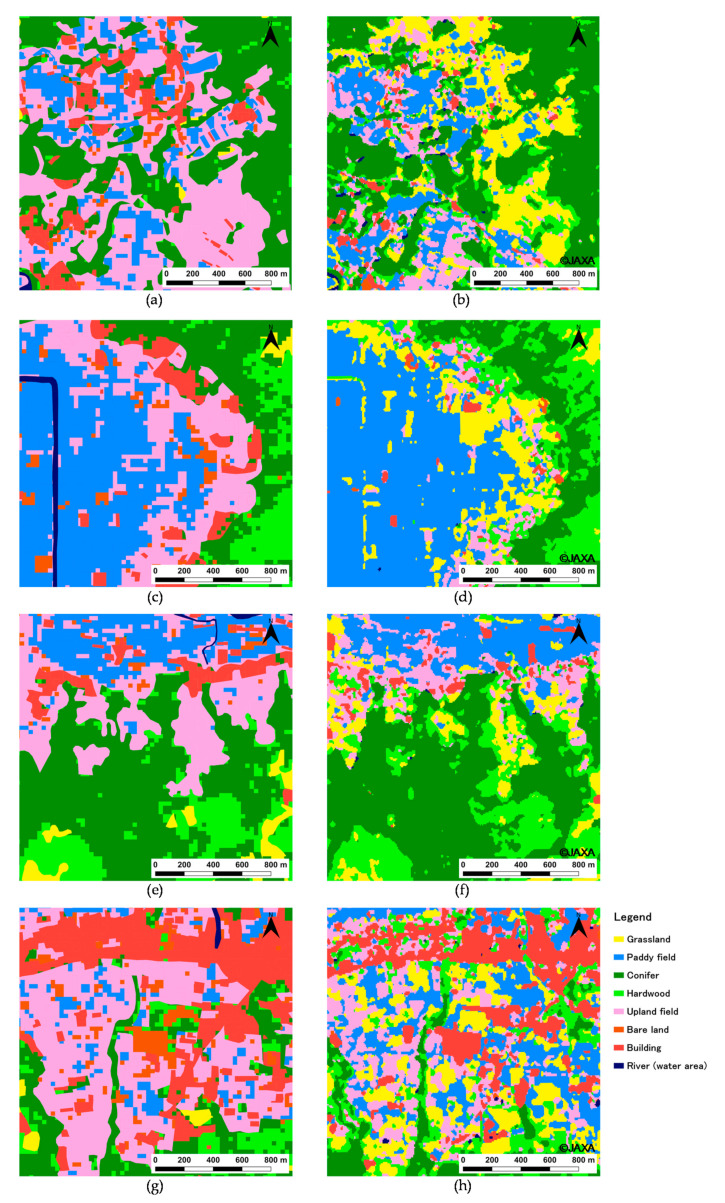
Comparison between the map from the analysis results for 2015 and JAXA’s map for 2006–2011 (ver16.09); (**a**) analysis results, (**b**) JAXA, (**c**) analysis results, (**d**) JAXA, (**e**) analysis results, (**f**) JAXA, (**g**) analysis results, (**h**) JAXA.

JAXA was born through the merger of three institutions, namely the Institute of Space and Astronautical Science (ISAS), the National Aerospace Laboratory of Japan (NAL) and the National Space Development Agency of Japan (NASDA) in 2003 [32]. It was designed as a core agency that supports space development and utilization of the Japanese government through its technologies. JAXA became a National Research and Development Agency in April 2015. JAXA is the largest in a National R&D Agency. High-resolution land use and land cover maps (Figure 3h,i) were published by the Advanced Land Observing Satellite (ALOS)/ALOS-2 Science Project and “Earth Observation Priority Research: Ecosystem Research Group” [11]. Classifications were performed by post-classification editing by applying algorithms such as a Bayesian classifier with Kernel Density Estimation (KDE) and prior probability estimation with KDE to some data, such as ALOS data, Landsat data, 10 m resolution digital elevation data, and information on training data from the Site-based dataset for Assessment of Changing Land cover by JAXA (SACLAJ) database. Depending on the map, the applied algorithms and dataset are different. Details can be confirmed on the homepage [11]. As a result of accuracy verification of the newest map (ver18.03), using 3000 points of reference data, which were independently obtained from the SACLAJ database (separately from the training data), the overall accuracy is 81.6% [11]. However, in our research area, land cover is significantly different from the actual land cover, which we confirmed by field survey (Figure 3i). In particular, misclassifications of the distribution of paddy fields in Nango-Dani and grasslands in the central cone stand out. In addition, upland fields are not distributed around the central cone. On the other hand, Figure 3h shows that the match between the actual land cover and vegetation map (Figure 3g; vg67) is superior compared with Figure 3i. As a result of accuracy verification of ver 16.09 data using 1409 points of reference data, which were independently obtained from the SACLAJ database (separately from the training data), the overall accuracy is 78.0% [11]. However, the accuracy of the classification for upland fields is 45.2%. Half of the upland fields were misclassified into grasslands. This misclassification can be confirmed in our research area (Figure 5b,d,f,h). Figure 5 shows the extracted the parts in which the misclassifications are remarkable. In our classification map (Figure 5a,c,e,g), the area of grasslands in the JAXA map is well classified as upland fields. Additionally, upland fields were misclassified into hardwoods in the JAXA map (Figure 5b,d,f,h). The percentage of misclassification for hardwoods is 6.7% [11]. These misclassifications are a serious issue because we are aiming to determine the relationship between spring water and transformations of grasslands into forests. When the NDVI was used independently, paddy and upland fields appeared around the central cone and outer rim. Thus, GIS data was particularly effective to correct the area of these fields in this study. In other words, although it is a simple and conventional classification method that uses the NDVI time series, the land cover in Aso caldera is well expressed by combining GIS data. The GIS data (vg67 and vg3) which we used in this study are unique to Japan. However, our method to classify agricultural fields excluding damaged paddy fields is versatile because agricultural fields can be easily distinguished in a high-resolution satellite image such as SPOT and the GIS data can be created by ourselves.

### 3.2. Land Cover Change

**Figure 6:**
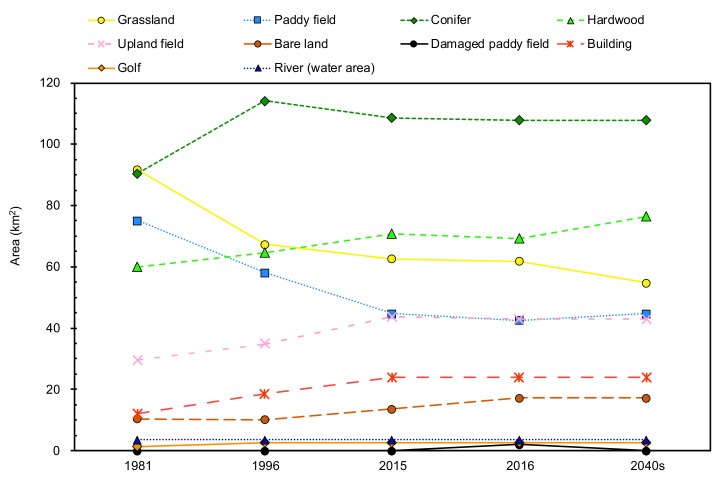
Temporal changes in the area for 10 categories.

Figure 6 shows the secular change in area in each land cover category. The changes between 1981 and 2015 are significantly represented in the dominant land covers: grasslands, conifers, hardwoods, paddy fields, and upland fields. Grasslands, which occupied the largest area in 1981, significantly decreased from 91.6 km^2^ to 62.6 km^2^. The transformations around the central cones and northeastern part of the outer rim are notable (Figure 3a–c). The grasslands in Aso caldera are ‘semi-natural grasslands’ that have been maintained by preventing their transition to forests due to artificial disturbances such as open burning, grazing, and mowing [33]. Since 1965, the grassland area that lacks artificial disturbances due to a variety of factors, such as the aging of farmers, the lack of successors, changes and stagnation in the agriculture and livestock industry, and changes in lifestyle, has been increasing [33]. Under these social conditions, semi-natural grasslands in Aso caldera transition to forests [33]. Therefore, these results indicate that the increase in the hardwood area from 60.0 km^2^ to 70.7 km^2^ is due to the transition of grasslands. In addition, grasslands have been converted to planted forests, such as Japanese cedar and cypress [34], increasing the area of conifers from 90.4 km^2^ to 108.6 km^2^. However, the conifers did not continue to increase until 2016 and decreased slightly between 1996 and 2015. It is probable that this decrease was caused by trimming, according to the satellite images. In 2016, a number of landslides due to the earthquake occurred on a hillside where forests and grasslands are distributed. The landslides that occurred near the confluence of two rivers (western part of Aso caldera) were the largest (Figure 3d). The affected area was about 200 m in width, 5–10 m in depth, and 325 m in height [35]. Because of the impacts of landslides, the areas of grasslands, conifers, and hardwoods were reduced by 0.8 km^2^, 0.7 km^2^, and 1.5 km^2^, respectively. As a result, the area of grasslands in 2016 decreased to 68% of that in 1981. There is an ongoing situation in which grasslands cannot be maintained, especially in Nangou-Dani, because of earthquake-induced damages such as road disconnection. According to the future prediction, the area of grasslands in 2016 could decrease by about 7.2 km^2^ by the 2040s, reaching 60% of the area in 1981. The transformation is remarkable in the western part of the outer rim in Nangou-Dani (Figure 3e).

Although paddy fields occupied the third-largest area in 1981, it drastically decreased by 2015 as a result of the social factors, which are different from that in the grasslands. The Japanese government implemented the rice production adjustment policy that encouraged the cultivation of crops other than rice because the demand for rice had decreased as a result of changes in consumer tastes since the 1970s [36]. Thus, the area of paddy fields decreased from 75.1 km^2^ to 44.7 km^2^ by 2015, which is a larger reduction than that of grasslands. In particular, the paddy fields distributed on the foot of the central cone and outer rim changed to upland fields (Figure 3a–c). At such locations, there is no spring or river water to use for paddy fields. Thus, groundwater needs to be pumped up, which costs money, so farmers have apparently converted crops in line with the policy goals. On the other hand, because the paddy fields were converted to upland fields in response to the policy, the area increased from 29.5 km^2^ to 43.6 km^2^. However, as mentioned above, as the upland fields in 2016 include abandoned fields, the obtained value is larger than the actual cultivated area. In 2016, damaged paddy fields appeared as an impact of the earthquake. That caused a decrease in the paddy field area, which, in 2016, reached 57% of the area in 1981. The damaged paddy fields are mainly distributed in Aso-Dani around earthquake faults (Figure 1 and Figure 3d). The area of damaged paddy fields in Aso-Dani was larger than that in Nangou-Dani. In this study, we assumed that the damaged paddy fields will have recovered in the 2040s. Thus, the area of paddy fields is expected to increase from the value in 2016. Although the decrease in paddy fields caused by the change in crops will disappear because the reduction policy was abolished in 2018, it may decrease in the future owing to the aging of farmers.

The area of buildings has been slightly increasing, although the change rate is much lower than that of dominant land covers. The total population of Aso City, Minamiaso Village, and Takamori Town was 55,820 in 1980, 51,931 in 1995, and 44,846 in 2015, respectively, indicating a decreasing trend [37]. Although these municipalities are also distributed outside the Shirakawa and Kurokawa River watershed, it is possible that the population in the areas of analysis is also declining because the central administrative center is located in Aso caldera. Although the population has a decreasing trend, the increase in houses is remarkable. In addition to agriculture, tourism is the most active industry in Aso caldera. The number of tourists in Kumamoto prefecture has been increasing every year since 1988 [38]. The Aso region is no exception. Aso Farm Land, which was built in 1995 and is one of the largest theme parks in Japan, attracts millions of visitors each year. The number of workers in the tertiary industry has increased from 12,591 in 1981 to 15,287 in 2006. Therefore, the increase in building area must be mainly due to the increase in commercial facilities. Such urbanization is one of the factors of the decrease in dominant land covers that play an important role in groundwater recharge. However, buildings in residential areas are not predicted to increase in the future since the population has been decreasing. Magnificent natural settings, such as the grasslands and spring water of Nango-Dani, are important tourism resources. There are also foreign tourists who aim to view these types of natural environments. Thus, decreases in grasslands and spring water mean a loss of tourism resources, which can result in a decrease in the number of tourists. As a result, tourism facilities are no longer needed, in which case, the building area will not increase in the future.

### 3.3. Potential Groundwater Recharge

**Figure 7:**
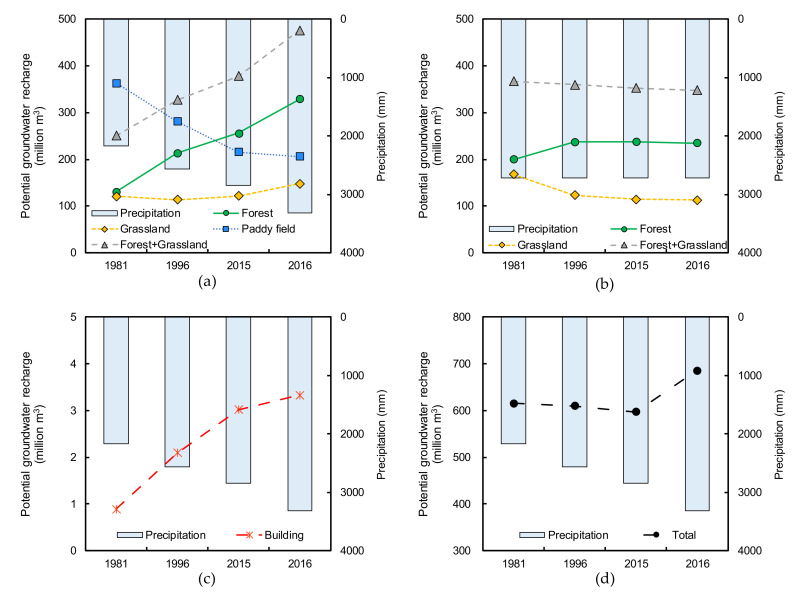
Temporal changes in potential groundwater recharge for Forest, Grassland, Paddy field, and Building: (**a**) actual precipitation, (**b**) constant precipitation, (**c**) actual precipitation, (**d**) actual precipitation.

Figure 7a shows the estimated temporal change in groundwater recharge from 1981 to 2016. The groundwater recharge in the grasslands was about 121 million m^3^ in 1981. It decreased to 114 million m^3^ in 1996, corresponding to the decrease in grasslands. Furthermore, while the area of grasslands decreased from 1996 to 2015 and 2016, the groundwater recharge increased to 122 million m^3^ in 2015 and 148 million m^3^ in 2016. Although precipitation of 2165 mm in 1981 increased to 2570 mm in 1996, the groundwater recharge decreased, indicating that the area change in grasslands was a predominant factor. However, the precipitation further increased to 2844 mm in 2015 and 3318 mm in 2016; consequently, the precipitation became a dominant factor that resulted in an increase in groundwater recharge in the grasslands. In 2016, the groundwater recharge in forests (conifers and hardwoods) was more than double that of 1981 due to the synergistic effect of increased area and increased precipitation. The temporal change in total groundwater recharge in forests + grasslands is similar to that in forests because the groundwater recharge in forests is much higher than that in grasslands. In other words, the change in groundwater recharge induced by the change in each area was not well represented because the annual precipitation occasionally increased in the analyzed year. This result is not appropriate for evaluating the impact of land cover changes on groundwater recharge. Therefore, the groundwater recharge was recalculated using constant precipitation (Figure 7b). The applied precipitation is the average of the analyzed years. Since the precipitation is constant, the change in groundwater recharge naturally depends on the area, representing the groundwater recharge decreases in grasslands and increases in forests. The groundwater recharge shows an overall decreasing trend because the decrease in recharge in grasslands is greater than its increase in forests. In other words, the groundwater recharge decreases because of the change of grasslands to forests. This is mainly because forests have higher evapotranspiration than grasslands.

Groundwater recharge from the paddy fields in 1981 was about 363 million m^3^ (Figure 7a). After 1981, with the reduction in the area due to the rice production adjustment policy (Figure 6), groundwater recharge decreased to 216 million m^3^ in 2015. Then, it further decreased to 206 million m^3^ because some paddy fields were damaged by the 2016 Kumamoto earthquake. The area of paddy fields is smaller than that of forests, which is the largest land cover (Figure 6), but in each year, groundwater recharge exceeds at least 200 million m^3^ without precipitation. This is because paddy fields have higher infiltration rates [27], which indicates again that these areas are important for groundwater recharge. Groundwater recharge from the building area increased from 0.9 million m^3^ in 1981 to 3.3 million m^3^ in 2016 (Figure 7c). This is due to an increase in both area and precipitation. Although the area of buildings is 10–20 km^2^ (Figure 6), groundwater recharge is much smaller than that of the other land covers since most of the precipitation flows out as surface runoff. The total groundwater recharge from four land covers is shown in Figure 7d. Although the decrease in groundwater recharge due to the change of grasslands to forests is not well represented in the actual precipitation (Figure 7a), total groundwater recharge decreased between 1981 and 2015 because the decrease in paddy fields became a dominant factor. In 2016, the precipitation became dominant, and then the total groundwater recharge increased. Therefore, focusing on only these analysis years, the main cause of the decrease in spring water is likely the decrease in paddy fields.

## 4. Conclusions

In this study, in order to obtain important data for building a water circulation model, we generated land cover maps from the past to the present and the future by integrating the NDVI time series and GIS data. The generated land cover maps were shown to have sufficient validity by comparing them with vegetation maps (vg67 and vg3) and the account book of paddy fields. Compared generated land cover map with the JAXA map, the paddy and upland fields in our classification were particularly well corrected by using the GIS data, indicating that the GIS data can improve a simple and conventional classification method that uses the NDVI time series. The area of each category reflected social factors, such as the Japanese government’s rice production adjustment policy, the stopping of burning grasslands, tree plantation, and active commerce. In particular, changes in the land cover in Aso caldera were greatly affected by changes in agriculture. Evaluating the impacts of the transformation of grasslands to forests on groundwater recharge revealed that the annual precipitation in the analyzed year increased, so the change in groundwater recharge induced by the change in land cover was not well represented. However, when the precipitation remained constant in the analysis, the groundwater recharge decreased due to the transformations. In addition, paddy fields had a higher groundwater recharge capacity in Aso caldera, even though precipitation was ignored when paddy field agriculture was not practiced. These results provide important data to support the maintenance and conservation of grasslands and paddy fields in terms of groundwater conservation in Aso caldera. According to the total groundwater recharge, the main reason for the spring water decrease is likely the decrease in paddy fields. However, the land covers in places related to spring water are an important factor. In the future, we will attempt to build a water circulation model that includes all land covers and decipher the cause of the spring water decrease.

## Figures and Tables

**Table 1:** Landsat satellite images used in the study.

Year	Season I	Season II	Season III	Season IV	Season V
2017	19 February *				
2016		20 March *	23 May *	11 August *	30 October *
2015			21 May *		
1997		1 April **			
1996			1 June **	5 September **	
1982				31 July ***	
1981		23 March ****	3 June ****		

* Landsat-8 OLI, ** Landsat-5 TM, *** Landsat-3 MSS, **** Landsat-2 MSS.

**Table 2:** Red band and near-infrared band in Landsat-8, -5, and -2, 3.

Band	Landsat-8 OLI	Landsat-5 TM	Landsat-2, 3 MSS
Red band (*R*)	band-4	band-3	band-5
Near-infrared band (*NIR*)	band-5	band-4	band-6 and band-7
